# A pathological complete response after combined chemotherapy of gemcitabine and S-1 in advanced biliary tract cancer with para-aortic lymph nodes metastasis: a case report

**DOI:** 10.1186/s40792-017-0303-5

**Published:** 2017-02-12

**Authors:** Takeshi Watanabe, Junji Furuse, Naohiro Okano, Yutaka Suzuki, Hiroshi Kamma, Masanori Sugiyama

**Affiliations:** 1 Department of Surgery, Kyorin University School of Medicine, 6-20-2 Shinkawa, Mitaka, Tokyo 181-8611 Japan; 2 Department of Medical Oncology, Kyorin University School of Medicine, Tokyo, Japan; 3 Department of Pathology, Kyorin University School of Medicine, Tokyo, Japan

**Keywords:** Biliary tract cancer, Chemotherapy, Conversion surgery, Pathological complete response, Gemcitabine, S-1

## Abstract

**Background:**

Biliary tract cancer is a rare malignancy that carries poor prognosis. Complete surgical resection is the only curable treatment. However, biliary tract cancer patients are often diagnosed with advanced stages and treated in systemic chemotherapy or palliative treatment settings rather than curative surgery.

**Case presentation:**

This case report describes a pathological complete response of advanced biliary tract cancer achieved after 2 years of intensive combined chemotherapy with gemcitabine and S-1. A 70-year-old female patient who developed severe stenosis in the middle common bile duct with para-aortic lymph nodes swelling was diagnosed with advanced extrahepatic biliary tract cancer that includes metastatic para-aortic lymph nodes and treated with combined chemotherapy of gemcitabine and S-1. After 32 courses of the combined chemotherapy, substantial shrinkage of these enlarged lymph nodes were confirmed and she underwent pylorus-preserving pancreaticoduodenectomy. The pathological examination revealed no viable neoplastic cells in the common bile duct or lymph nodes. She did not receive any further adjuvant chemotherapy. No recurrent lesions have been detected for 48 months after the primary surgery.

**Conclusions:**

This case shed light on the probability of conversion and/or adjuvant surgery for biliary tract cancer with novel systemic chemotherapy regimens.

## Background

Biliary tract cancer (BTC) is rare and often aggressive malignancy. BTC includes four distinct categories of adenocarcinoma intrahepatic cholangiocarcinoma, extrahepatic cholangiocarcinoma, gallbladder cancer (GBC), and ampullary carcinoma. Complete surgical resection only offers patients a chance for cure. However, even if curative intent surgery is applied to resectable BTC patients, 5-year survival rates still remain low: 33.1% for bile duct cancer, 52.8% for ampullary cancer, and 41.6% for gallbladder cancer [1]. BTC patients are often diagnosed with advanced stages in which tumor metastasizes to distant organs or involves adjacent tissue and treated with systemic chemotherapy. Gemcitabine has been the mainstay of the systemic chemotherapy treatment of biliary tract cancer. Moreover, recent advances in development of chemotherapy regimens have gained additional survival benefits for advanced BTC patients. The combination of gemcitabine (GEM) and cisplatin (GC therapy) has begun to be considered as the standard of care for BTC treatment. Meanwhile, S-1 is often used as second-line treatment for advanced BTC that is refractory to GEM treatment. The use of the combined chemotherapy of GEM and S-1 for advanced BTC has been examined recently and shown favorable results. This case report describes that a pathological complete response was obtained by curative surgery after 32 courses of combined chemotherapy of GEM and S-1 for a patient who was diagnosed with extrahepatic biliary tract cancer with para-aortic lymph node metastasis.

## Case presentation

**Fig. 1:**
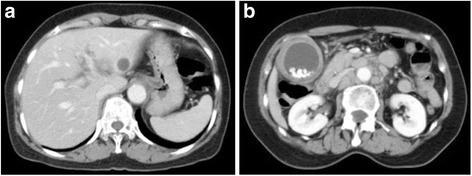
**a** An abdominal CT scan image shows dilatation of intrahepatic biliary duct. No mass lesions are seen in the liver. **b** A CT scan image shows multiple para-aortic lymph nodes swelling and multiple gallbladder stones with gallbladder wall thickness

**Fig. 2:**
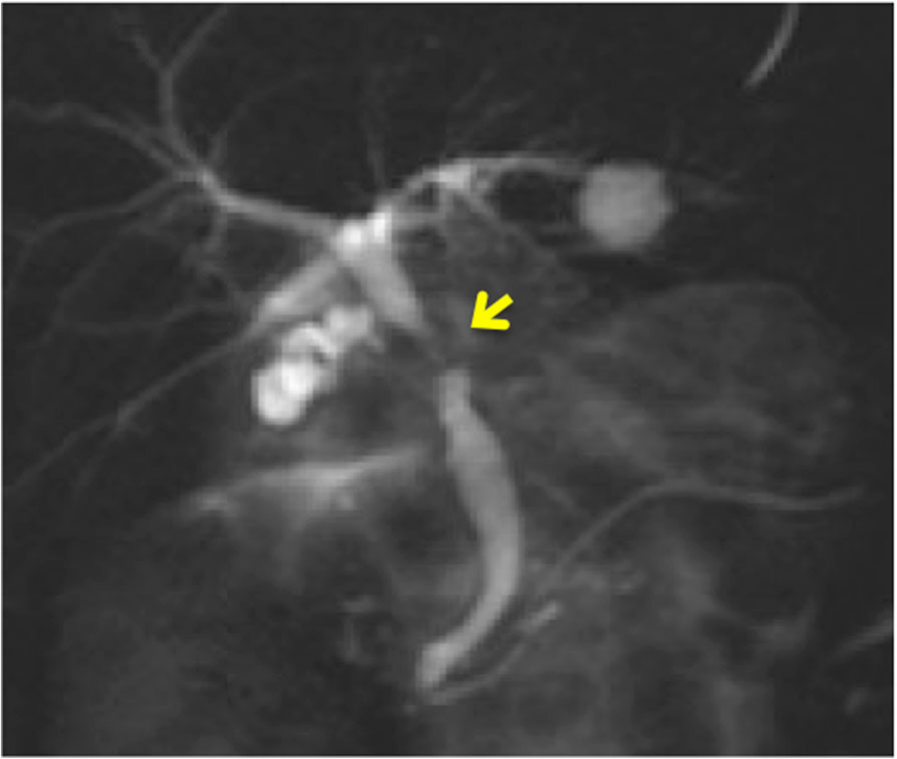
A MRCP image shows sever stenosis in the middle common bile duct (*arrow*). Gallbladder was not clearly visualized

**Fig. 3:**
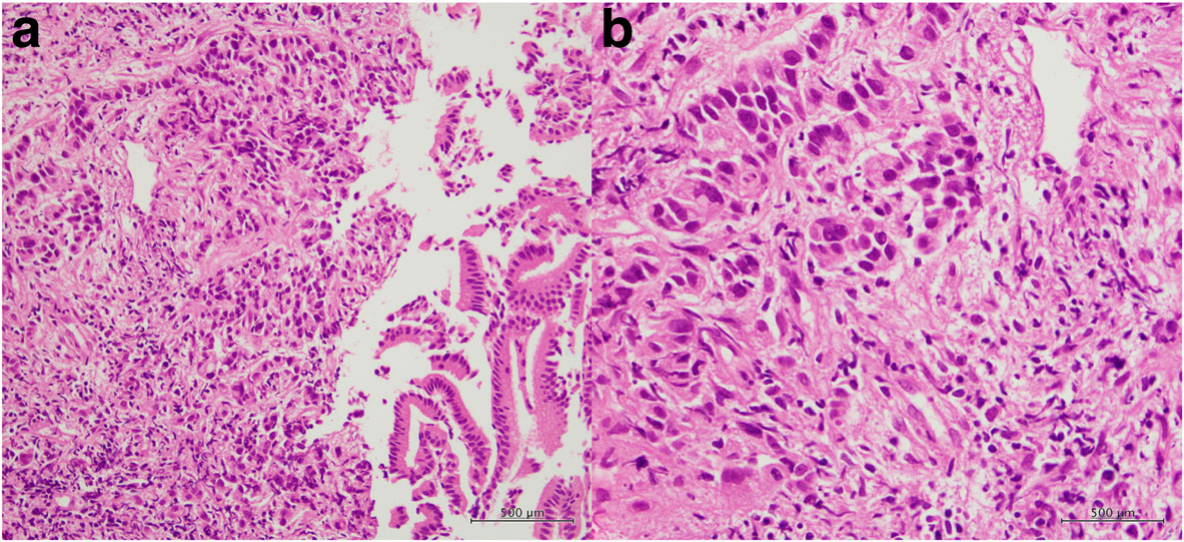
Endoscopic transpapillary bile duct biopsy specimen shows infiltration of poorly differentiated adenocarcinoma cells beyond muscularis mucosa **a** low power field image, **b** high power field image

A 70-year-old female patient was admitted to the hospital because of 2 weeks of persisting epigastric discomfort. She had medical history of untreated choledocolithiasis for 4 years. Physical examination was only remarkable for tenderness in the right upper quadrant area. The patient’s white blood cell count was 5500/μl, hemoglobin was 12.4 g/dl, and platelet count was 190,000/μl. Any disorders for liver function tests were not detected. Serum bilirubin level was also within normal range. Serum tumor marker values such as CEA and CA 19-9 were not elevated. However, CRP was elevated to 4.8 mg/dl. CT scan revealed dilatation of intra and extra hepatic bile ducts, gallbladder stones and gallbladder wall thickness. No mass lesions in the head of the pancreas or in the biliary tract were detected except for enlarged swollen intra abdominal and para-aortic lymph nodes (Fig. 1a, b). Given these findings, she was initially diagnosed with acute cholecystitis. Although percutaneous transhepatic gallbladder drainage (PTGBD) was safely performed, common bile duct was not radiographically visualized. Magnetic response cholangiopancreatograpy (MRCP) showed severe stenosis in the middle extrahepatic bile duct with dilatation of the intrahepatic bile ducts (Fig. 2). Histological diagnosis of adenocarcinoma of the middle extrahepatic bile duct was made by endoscopic transpapillary bile duct biopsy (Fig. 3). A covered self-expandable metallic stent (ZENOSTENT covered 10 mm × 80 mm; Zeon Medical Inc., Tokyo, Japan) was placed endoscopically. Based on these findings, she was diagnosed with BTC with para-aortic lymphnodes metastasis and treated with a combined chemotherapy regimen of GEM and S**-**1. 1000 mg/m^2^ of GEM was intravenously administered at day 1 and 8, and 80 mg/day of S-1 was orally given for 14 days followed by 7 days of rest, repeated every 3 weeks. After the six courses, enlarged para-aortic lymph nodes began to shrink. Adverse effects included grade 2 suppression of bone marrow function, hair loss, and GI symptoms such as diarrhea, and vomiting that were tolerable and managed by reducing administration dose to 80% of the initial dosage without discontinuation of the chemotherapy.

**Fig. 4:**
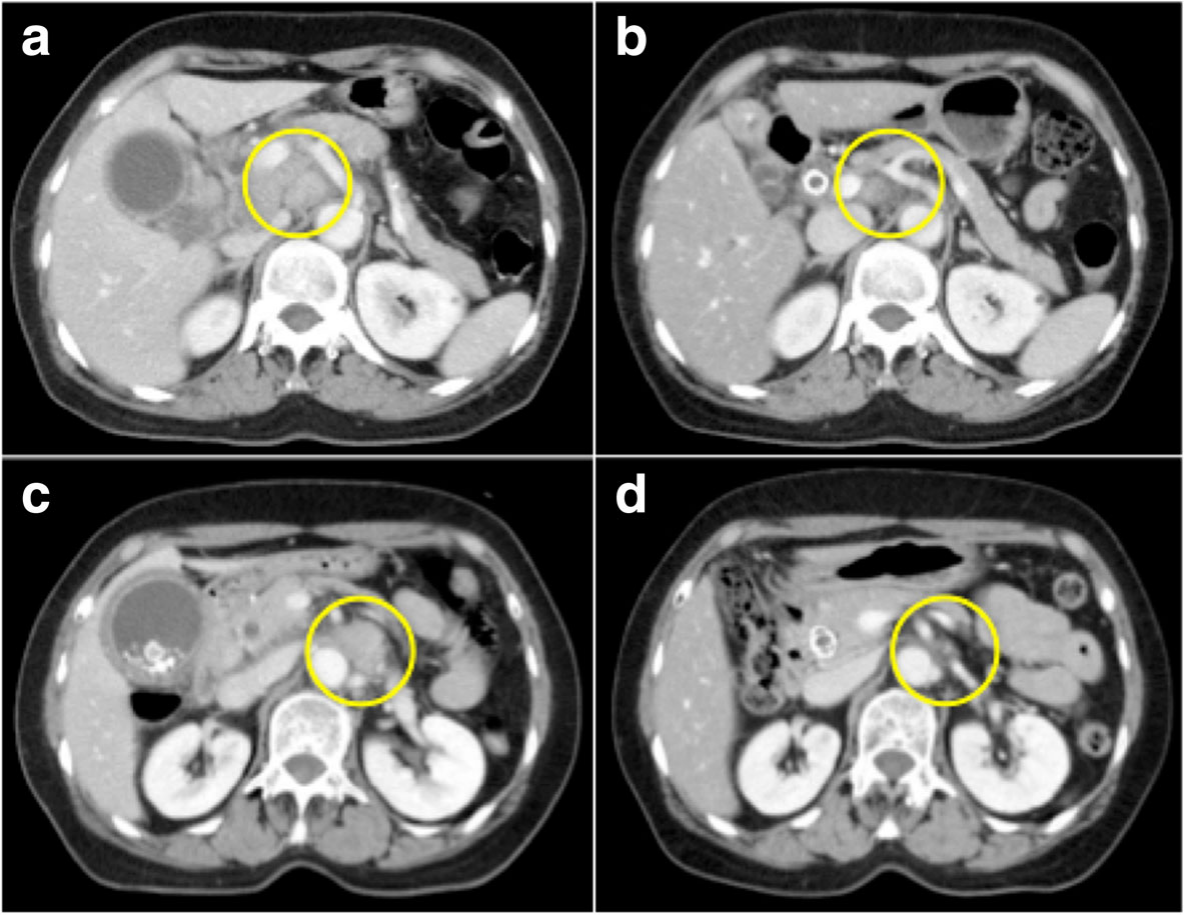
Abdominal CT images scan showing swollen regional lymph nodes before chemotherapy (**a**, **b**), and after 32 courses of chemotherapy (**c**, **d**). Maximal diameter measurements of the regional lymph nodes were decreased by more than 50%: from 20 mm before chemotherapy (**a**) to 5 mm after chemotherapy (**c**) for celiac lymph node #9 and from 20 mm before chemotherapy (**b**) to 8 mm after chemotherapy (**d**) for para-aortic lymph node #16a2 inter

**Fig. 5:**
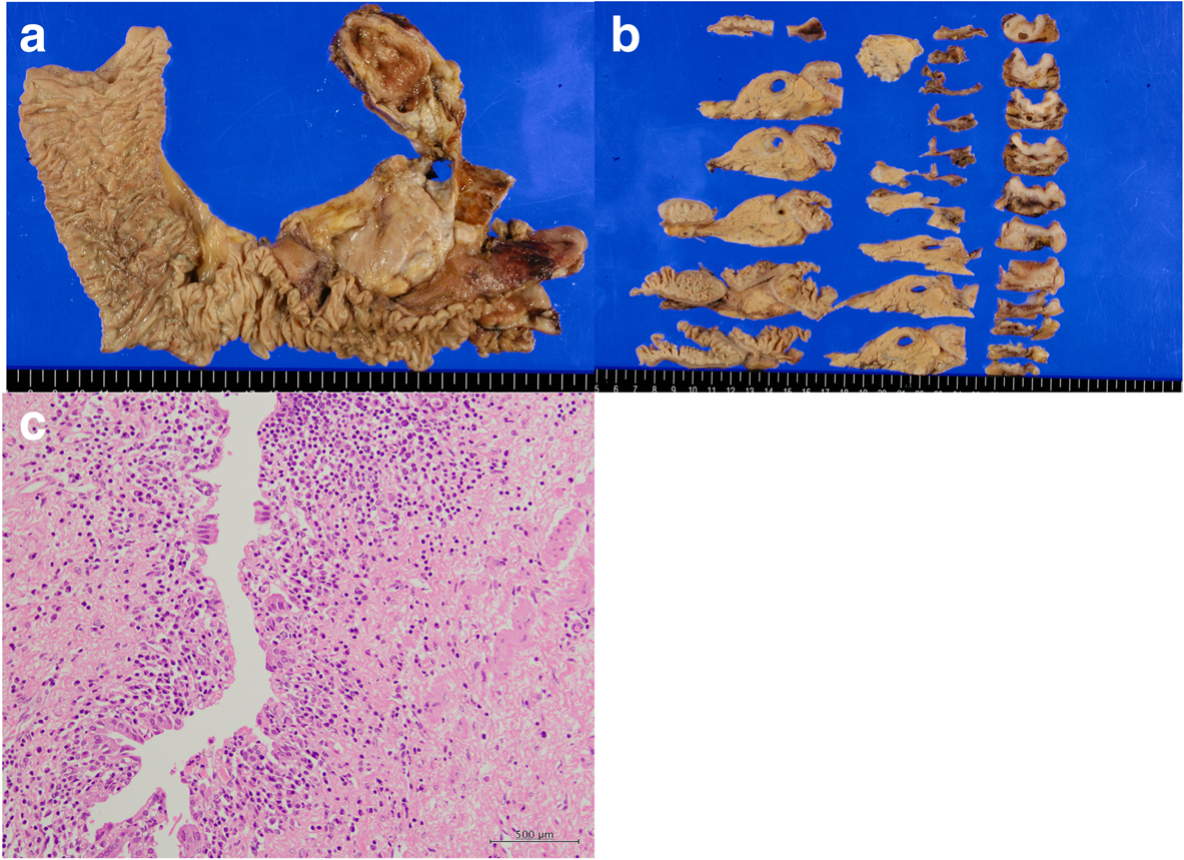
Macrographs of the resected specimen show erosion of biliary mucosa and disappearance of biliary mucosa cells in the middle common bile duct (**a**, **b**). Histopathological examination reveals no residual cancer cells in the middle common bile duct (**c**)

**Fig. 6:**
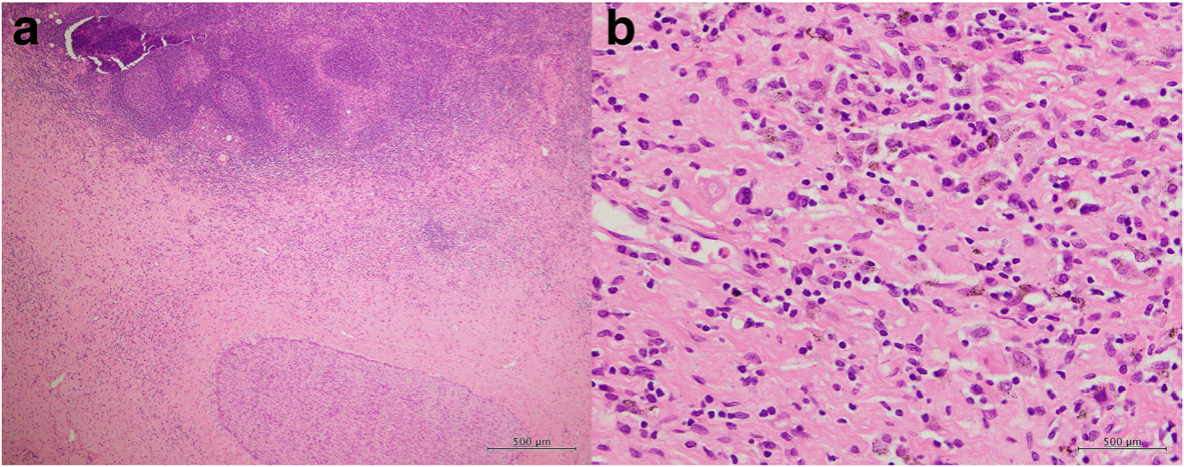
Resected lymph node shows fibrosis with histiocyte infiltration (**a**, **b**)

Finally after 32 courses of the combined chemotherapy, many enlarged lymph nodes were reduced by more than 50% in diameter (Fig. 4a, b). The patient agreed to undergo curative intent surgery 2 months after the final dose administration. Peritoneal lavage cytology did not detect any adenocarcinoma cells. Intra operative frozen section analysis of para-aortic lymph node station 16b1 did not detect any tumor cells, either. Therefore, pylorus-preserving pancreaticoduodenectomy was safely performed. Hepatic duct was transected at the level of 1.0 cm below the union of left hepatic duct and right hepatic duct. Twenty-seven lymph nodes including stations 6, 8a, 12a, 12p, 13a, 13b, 16inter, 16b1, and17a were harvested. Pathological examination of the resected specimen showed no residual neoplastic cells in bile duct, cystic duct, and gallbladder. Fibrosis in the bile duct with the presence of plasma cells and macrophages was observed (Fig. 5a–c). Para-aortic lymph node specimens also showed replacement fibrosis and aggregates of macrophages, but did not show any residual tumor cells. (Fig. 6a, b). Postoperative complications included grade B pancreatic fistula that was treated conservatively. The patient was discharged on postoperative day 45. The patient did not agree to receive adjuvant chemotherapy. 48 months after the surgery, the patient is in a good condition, and no suspicious recurrent lesions have been detected.

## Discussion

For those whose stage is beyond the scope of curative surgery, systemic chemotherapy is chosen to apply. GEM has achieved improved response rates and survival benefit since its approval for BTC treatment [2–4]. For example, GEM-based chemotherapy such as GC therapy achieves good antitumor activity. Recent results from a multicenter, randomized phase III trial, the UK ABC-02 trial that evaluated GEM with or without cisplatin in patients with advanced or metastatic BTC have demonstrated a clear survival advantage (hazard ratio 0.68, *P* = 0.002) for the combination of GEM and cisplatin (GC regimen) without added clinically intolerable toxicity [5]. A Japanese multicenter phase III trial using the same regimen has also shown favorable results consistent with the result of ABC-02 study: the median survival and overall response rate of GC vs. GEM alone were 11.2 vs. 7.7 months and 19.5 vs. 11.9%, respectively [6]. Along with these results, GC regimen has been considered as new standard regimen for advanced BTC. Meanwhile, S-1 is an oral fluoropyrimidine anticancer drug designed to enhance the anticancer activity of fluorouracil and reduce its gastrointestinal toxicity [7]. S-1 monotherapy has already shown its anticancer effect against BTC with a 35% of response rate [8]. Based on this result, in 2013, a multicenter phase II study that compares GEM alone, and a combination chemotherapy with GEM and S-1 (GS regimen) was conducted, reporting that GS regimen showed a 36.4% response rate and mild toxicity for patients with advanced biliary tract cancer [9]. A comparison study between GS and GC regimens for advanced BTC is currently ongoing in a randomized setting (JCOG1113, UNIM000010667).

On the other hand, data is derived from small retrospective trials and case reports about neoadjuvant and conversion chemotherapy of BTC. Kato et al. revealed that GEM regimen is able to downsize tumors towards resectable stages and lead to produce more R0 resections than the control group [10]. A few cases of successful conversion chemotherapy from initially unresectable BTC to resectable have been reported [11–13]. No general consensus about this issue has been made.

The most astonishing point in this case report is the achievement of pCR both in the primary tumor lesion and metastatic lymph nodes, although preoperative diagnosis was not made. Preoperative CT evaluation detected 13 lymph nodes, maximum short axis diameter (MSAD) of which is larger than 10 mm. The shapes of these lymph nodes are irregular round rather than flat shape. MSAD of lymph node station 8a was 30 mm. Nodal enlargement of station 16b1 was also found. Although there is no agreement on the definition of the threshold of nodal enlargement for metastasis, findings such as total numbers of enlarged nodes, MSAD, and wide spread of nodal enlargements strongly suggested lymph node metastasis rather than nodal enragement caused by cholecystitis. Furthermore, the presence of scarring without tumor cells in the harvested lymph node specimen most likely represents complete response to chemotherapy. Previous reports addressed pathological complete response after chemotherapy for advanced BTC. In a single center phase II study that evaluated combination chemotherapy of GEM and oxaliplatin (OXIGEM) for advanced GBC patients, Sharma et al. [14] reported one pCR. Another case report also showed one pCR case of BTC with five courses of the OXIGEM regimen [15]. Walker et al. reported that a case of pCR for locally advanced common bile duct (CBD) cancer was achieved with GC regimen [16]. Lim et al. also reported that GS regimen produced pathological complete response for CBD cancer and radiographic disappearance of liver metastasis [17]. However, none of these reports achieved pCR of the metastatic lesion along with the primary lesion as described in this report. This patient was not initially treated in the neoadjuvant setting. Overwhelming response made her switch to undergo curative intent surgery after 2 years of the chemotherapy treatment. It might be possible that this patient had unique cancer profile and responded to the chemotherapy very well.

## Conclusions

We experienced a patient with advanced BTC who was treated with GEM plus S-1 and achieved pCR. GS regimen may be applicable for advanced BTC. Further cases treated with GS regimen are needed to evaluate its efficacy as conversion chemotherapy regimen for advanced and unresectable BTC.

## Abbreviations

BTC: Biliary tract cancer
CA 19-9: Carbohydrate antigen 19-9
CBD: Common bile duct
CEA: Carcinoembryonic antigen
CRP: C-reactive protein
CT: Computed tomography
GC: Gemcitabine and cisplatin
GEM: Gemcitabine
GS: Gemcitabine and S-1
JCOG: Japan Clinical Oncology Group
MRCP: Magnetic response cholangiopancreatography
MSAD: The maximum short axis diameter
OR: Odds ratio
pCR: Pathologic complete response
PTGBD: Percutaneous transhepatic gallbladder drainage
